# Iron Neurotoxicity and Protection by Deferoxamine in Intracerebral Hemorrhage

**DOI:** 10.3389/fnmol.2022.927334

**Published:** 2022-06-16

**Authors:** Zhe Li, Yang Liu, Ruixue Wei, Suliman Khan, Ruiyi Zhang, Yan Zhang, Voon Wee Yong, Mengzhou Xue

**Affiliations:** ^1^Department of Cerebrovascular Diseases, The Second Affiliated Hospital of Zhengzhou University, Zhengzhou, China; ^2^Academy of Medical Science, Zhengzhou University, Zhengzhou, China; ^3^Henan Medical Key Laboratory of Translational Cerebrovascular Diseases, Zhengzhou, China; ^4^Department of Clinical Neurosciences, Hotchkiss Brain Institute, University of Calgary, Calgary, AB, Canada

**Keywords:** intracerebral hemorrhage, brain edema, deferoxamine, iron overload, neuronal death

## Abstract

Intracerebral hemorrhage (ICH) is a subtype of stroke that is characterized by high morbidity and mortality, for which clinical outcome remains poor. An extensive literature indicates that the release of ferrous iron from ruptured erythrocytes in the hematoma is a key pathogenic factor in ICH-induced brain injury. Deferoxamine is an FDA-approved iron chelator that has the capacity to penetrate the blood-brain barrier after systemic administration and binds to iron. Previous animal studies have shown that deferoxamine attenuates ICH-induced brain edema, neuronal death, and neurological deficits. This review summarizes recent progress of the mechanisms by which deferoxamine may alleviate ICH and discusses further studies on its clinical utility.

## Introduction

Intracerebral hemorrhage (ICH) is caused by the rupture of blood vessels in the brain. It accounts for approximately 15% of all stroke types and affects about 2 million people worldwide each year (Gross et al., 2019). The prognosis of ICH is worse than that of the more common ischemic stroke; ICH has a 30-day mortality rate of 43–51%. Most patients that survive ICH have residual sequelae such as neurological dysfunctions (Krishnamurthi et al., 2020). The adverse outcomes associated with ICH are due to a combination of various pathophysiological processes, such as hematoma formation, space-occupying effects caused by the enlarging hematoma, local cerebral blood flow change, disruption of blood-brain barrier (BBB), and brain edema (Bai et al., 2020b; Chen et al., 2020; Zhang et al., 2021). The mechanism of brain injury after ICH can be broadly divided into primary injury caused by the mass effect of intraparenchymal hematoma, and secondary injury induced by neuroinflammatory responses and oxidative stress (OS) (Zhu et al., 2019; Xue and Yong, 2020; Holste et al., 2021).

Iron, as one of the main degradation products of hemoglobin catabolism, can cause secondary brain injury after ICH by promoting free radical formation and inflammatory responses (Zhu et al., 2021). Iron overload can lead to brain injury in several ways, such as lipid peroxidation and free radical formation (Stockwell et al., 2017). As an iron-chelating agent, deferoxamine can rapidly cross the blood-brain barrier (BBB) after systemic administration, and also effectively combine with iron ions to reduce the ferrous ion concentration in the hematoma area, thereby ameliorating the secondary neurological damage caused by ICH (Zeng et al., 2018). In ICH animal models, deferoxamine has been shown to be neuroprotective through several mechanisms, including reduction of hemoglobin-related edema and inhibition of neuronal death; it improves neurological deficits and brain atrophy after ICH (Cui et al., 2015; Zhang et al., 2022a). In this review, we summarize the mechanisms and advances of deferoxamine in the treatment of ICH.

## Erythrocyte Lysis and Brain Edema Formation

Notably, hemoglobin and its degradation products are neurotoxic and contribute to delayed neuronal injury and edema formation after ICH (Wagner et al., 2003; Hua et al., 2007). At present, the mechanisms of brain edema formation following ICH has not been fully clarified and it may occur through a series of pathophysiological processes, including platelet aggregation, clot formation, activation of the coagulation cascade, iron overload and hemoglobin toxicity, complement activation, secondary injury after reperfusion, and disruption of the blood-brain barrier (Appelboom et al., 2013; Wang et al., 2018). Studies have shown that clots begin to dissolve on the first day after ICH, and their by-products, including free hemoglobin, heme and free iron, contribute to brain injury that may last days or weeks (An et al., 2017).

**FIGURE 1:**
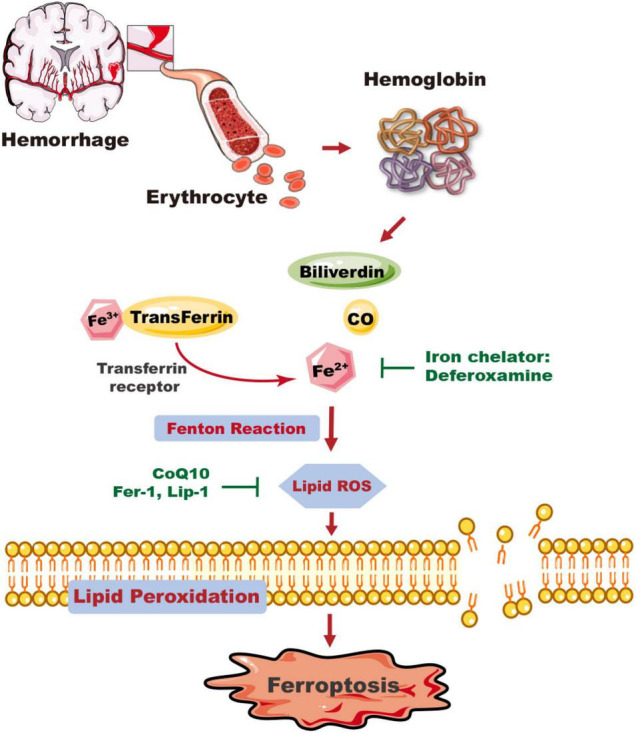
The mechanism of ICH-induced ferroptosis in the brain. Following ICH, erythrocytes release hemoglobin, which produces a degradation product, carbon monoxide (CO), biliverdin, and free iron. Excess Fe^2+^, the reactive form of iron, can generate ROS and cause membrane lipid peroxidation, and trigger ferroptosis. Deferoxamine can reduce the level of iron ions and inhibit the production of ROS and the occurrence of ferroptosis. The ferroptosis inhibitors CoQ10, Fer-1, and Lip-1 inhibit lipid ROS activity and ferroptosis. CO, carbon monoxide; CoQ10, coenzyme Q10; Fer-1, ferrostatin-1; Lip-1, liproxstatin-1.

Erythrocytes lyse after ICH and release large amounts of ferrous ions, which contribute to brain edema formation (Perez De La Ossa et al., 2010; Zhang et al., 2022d). In the rat model of autologous blood-induced ICH, brain edema peaks on the third or 4th day after the onset of ICH, and then resorbs and gradually diminishes over time (Xi et al., 1998). In the thromboplastic-induced rat model, the earliest peak of brain edema occurs within 48 h (Yang et al., 1994; Zhang et al., 2021). The potential toxic effects of erythrocyte degradation products have been demonstrated, and results indicate that early erythrocyte lysis in the hematoma facilitates brain injury including disruption of BBB, brain iron overload, and neurological deficits within 24 h in aged rats (Xue and Del Bigio, 2005; Balami and Buchan, 2012; Figure 1).

## Hemoglobin and Brain Injury

Hemoglobin and its degradation products are crucial factors in brain injury after ICH (Zhu et al., 2019). Heme oxygenase-1 (HO-1) is a cytoprotective enzyme that catalyzes the toxic heme into carbon monoxide (CO), biliverdin and ferrous iron (Wang and Dore, 2007). Biliverdin is further converted to bilirubin by biliverdin reductase, which possesses antioxidant activity. Heme oxygenase-1 is known to be upregulated in the brain (Schipper et al., 2019). In addition, zinc protoporphyrin (ZnPP), an inhibitor of HO-1, suppresses brain edema and neuronal damage (Zhou et al., 2014; Zhang et al., 2022d). Also, intraperitoneal injection of deferoxamine *in vivo* may minimize brain injury resulting from hemoglobin degradation. In conclusion, hemoglobin itself and its degradation products, especially iron, play a key role in secondary brain injury after ICH (Huang et al., 2002).

## Iron Triggers Neuroinflammation Following Intracerebral Hemorrhage

Iron and its homeostasis have been described as a hallmark of neuroinflammation (Wu et al., 2011), and mounting evidence supports that iron accumulation may lead to inflammatory responses (Wessling-Resnick, 2010; Bai et al., 2020a). It has been recognized that iron triggers a cascade of deleterious events, including activating microglia/macrophages, and elevating the levels of nitric oxide (NO) and reactive oxygen species (ROS) (Zhang et al., 2022b), matrix metalloproteinases (MMPs), tumor necrosis factor-α (TNF-α) and other inflammatory molecules, exacerbating neuroinflammation (Wu et al., 2002; Yong et al., 2019; Xue and Yong, 2020; Zhu et al., 2021).

The role of macrophages in iron homeostasis has been studied. The results show that proinflammatory microglia/macrophages tend to sequester iron, whereas regulatory microglia/macrophages express genes that promote iron release (Yong et al., 2019). Thus, neuroinflammation and iron are tightly linked, as the inflammatory environment is associated with iron accumulation and neuroinflammation following ICH may affect normal function throughout the entire brain (Ke and Ming Qian, 2003; Zhang et al., 2015; Li et al., 2016).

## Iron Triggers Neuronal Death Following Intracerebral Hemorrhage

Iron, as a trace element, is essential for the maintenance of normal cellular physiological functions. It is also involved in the synthesis of myelin and various neurotransmitters in the central nervous system (Xiong et al., 2014), while the dysregulation of iron homeostasis in the brain (iron overload or deficiency) can contribute to multiple brain injuries, including hemorrhagic and ischemic stroke (Garton et al., 2016). Excess iron generates large amounts of ROS mainly through the Fenton reaction, triggering inflammation and neuronal death, which can lead to long-term cognitive dysfunction and early brain edema (Bai et al., 2020c; Zhang et al., 2022c). Upon erythrocyte lysis, the concentration of iron persists at a relatively high level. In a rat model of ICH, the concentration of non-heme iron increases threefold and is sustained at a high level for 28 days (Hua et al., 2006).

Iron overload contributes to brain edema and iron chelators can attenuate the degree of hematoma-induced brain edema (Qing et al., 2009). Countering the iron-induced phospholipid peroxidation and DNA damage (Gu et al., 2011), deferoxamine ameliorates neurological deficits, reduces the area of brain edema and inhibits neuronal apoptosis after ICH in rats (Nakamura et al., 2003).

## Brain Iron Metabolism After Intracerebral Hemorrhage

The brain needs iron for its own metabolism and dysfunction occurs when iron homeostasis is imbalanced (Ke and Qian, 2007). The iron in the central nervous system is mainly transported through the BBB, while another part of iron is directly absorbed by brain cells. Iron transport across the BBB is mostly through the transferrin–transferrin receptor system (Tf-TfR) (Chiueh, 2001). As a plasma glycoprotein, transferrin binds to its receptor and plays an indispensable role in the transfer of iron. In addition, the blood cerebrospinal fluid (CSF) barrier has similar characteristics to the BBB regarding iron transport, which may be another important pathway for iron to enter the brain through the choroid plexus (Nnah and Wessling-Resnick, 2018). After being transported across the BBB or blood-CSF barrier, iron can rapidly bind to Tf secreted by oligodendrocytes and choroid plexus epithelial cells, thus facilitating uptake by brain cells (Bradbury, 1997). In the brain, ferritin is an iron storage-protein, expressed mainly in neurons and glial cells, consisting of a heavy chain (H-ferritin) and a light chain (L-ferritin), respectively (Connor et al., 2001). Ferritin plays a pivotal role in iron storage and maintenance of intracellular iron homeostasis (Yang et al., 2021).

## Deferoxamine Reduces Brain Injury After Intracerebral Hemorrhage

Deferoxamine is an iron chelator used clinically to treat severe anemia and iron overload disorders caused by repeated blood transfusions (Selim, 2009). Deferoxamine has a high affinity for Fe^3+^ and binds directly to iron in plasma and tissues, contributing to the formation of a stable, non-toxic, water-soluble complex. The subsequent excretion of the complex from the body thereby protects brain against iron overload (Zeng et al., 2018).

**FIGURE 2:**
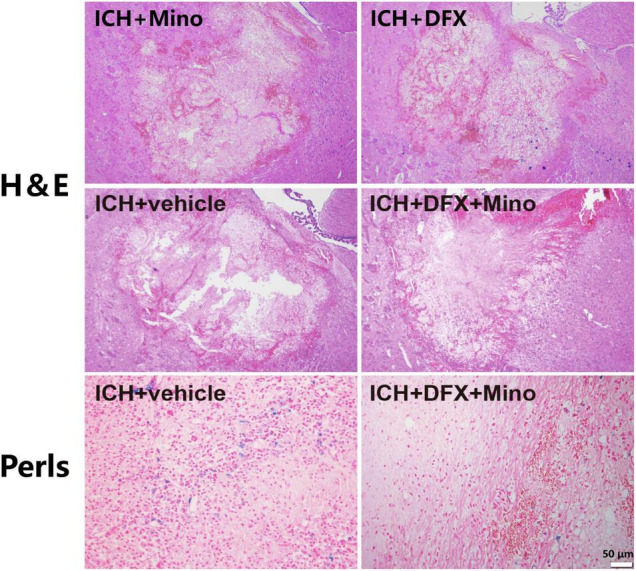
The combined therapy of deferoxamine and minocycline attenuated iron deposition and reduced the area of brain damage after ICH. Paraffin sections from rats at day 3 after ICH were analyzed, and representative images of Hematoxylin and Eosin staining showing the brain damage area, and Perls’ blue staining to reveal iron-labeled cells, are displayed. Scale bar, 50 μm.

After systemic administration, deferoxamine can penetrate the BBB, reduce iron content around the hematoma site, and thus attenuate secondary injury after ICH (Wan et al., 2019). As well, deferoxamine can catalyze the degradation of hemoglobin by increasing the expression of HO-1, thereby protecting against the toxic effects of free heme released during hemoglobin degradation mediated by glutamate (Schipper et al., 2019). Additionally, deferoxamine attenuated ICH-induced CD163 upregulation and brain cell death *in vivo*, and hemoglobin-induced CD163 elevation and neuronal death *in vitro* (Liu et al., 2017). Moreover, deferoxamine may reduce the production of reactive hydroxyl radical by inhibiting the Fenton and Haber—Weiss reactions (Matsuki et al., 1999). Our recent study found that the combination of deferoxamine with minocycline provided prominent neuroprotection after ICH (Sheng et al., 2018). Minocycline, a broad-spectrum tetracycline, can provide neuroprotection through its anti-inflammatory properties, including inhibiting microglia activity and reducing the expression of matrix metalloproteinases (Xue et al., 2010; Zhang et al., 2022a). Minocycline has also iron chelation capacity (Zhao et al., 2016; Liu et al., 2021). An obvious neurovascular protective effect by minocycline was observed in ICH models in rats (Lee et al., 2003). Our study revealed that the extent of brain damage, neuronal death, and the activation of microglia/macrophages were significantly reduced after ICH in minocycline and deferoxamine combined treatment. There was also decreased iron accumulation in the area around the hematoma in the combination treatment group (Figure 2), and animals recovered from their neurological deficits better (Li et al., 2021).

## Progress in the Translational Research of Deferoxamine in Intracerebral Hemorrhage

The efficacy of deferoxamine in treating hemorrhage-induced brain damage in animal models has been previously summarized (Selim, 2009; Cui et al., 2015; Zeng et al., 2018; Hu et al., 2019). Deferoxamine reduced the effects of ischemic and hypoxic brain injury, particularly in the cortex and hippocampus, and attenuated ICH-induced brain atrophy and neurological deficits (Okauchi et al., 2010; Hatakeyama et al., 2013). In the collagenase-induced ICH in mice, systemic administration of deferoxamine lowered local iron-deposition, inhibited secondary inflammation, reduced neuronal cell death, and improved recovery from neurofunctional deficits (Okauchi et al., 2009). Other studies have shown that deferoxamine diminished parenchymal iron levels but failed to attenuate functional impairment or lesion volume after ICH (Auriat et al., 2012). In the piglet model of ICH, deferoxamine treatment reduced the number of iron-positive cells, neuronal death, and iron accumulation around the hematoma (Gu et al., 2009; Hu et al., 2019). Similarly, Xie et al. showed that deferoxamine decreased white matter edema and TNF-α levels after ICH in piglets, both of which are supportive of deferoxamine as a potentially effective therapeutic agent for ICH patients (Xie et al., 2014). Our study demonstrated that deferoxamine reduced neuronal death, suppressed the activation of microglia/macrophages, decreased iron accumulation around the hematoma, lessened the area of brain injury, and improved neurological deficits in ICH (Li et al., 2021).

Deferoxamine is being tested in human clinical trials. A phase I multicenter, multi-quantitative trial was conducted to assess the tolerability and safety of deferoxamine in ICH (Selim et al., 2011). Deferoxamine was administered for 3 consecutive days by intravenous infusion starting within 18 h after the onset of ICH. The results found deferoxamine to be well tolerated in patients with ICH and was not associated with serious adverse events or mortality. A phase II clinical trial (NCT 01662895) was subsequently conducted (Selim et al., 2019). This placebo-controlled, randomized, double-blind trial was designed to investigate whether high-dose deferoxamine was effective in improving neurological function in patients with ICH, and to assess whether the drug should be investigated in a phase III trial. The results show that deferoxamine was safe and improved the chances of a good clinical outcome. The recent i-DEF trial (Intracerebral Hemorrhage Deferoxamine Trial) assessed modified Rankin Score (mRS) longitudinally by following ICH patients from day 7 to the end of the 6-month (Foster et al., 2022). The results revealed that a large proportion of patients continued to improve up to 6 months after ICH, signaling that deferoxamine may accelerate and alter the trajectory of recovery as assessed by mRS. Nevertheless, deferoxamine caused a series of adverse reactions, including allergic reactions, systemic allergies, and cardiovascular, hematologic, and neurotoxic effects (Yeatts et al., 2013). In addition, deferoxamine may increase cytotoxicity by inhibiting DNA synthesis (Liachenko et al., 2003).

## Future Directions

Encouraging, over the past decade, an increasing body of knowledge gained from basic science research on ICH has advanced considerations of therapeutic options. Targeting iron is one of this translation, with the appreciation that iron plays an important role in the formation of hematoma, neuronal death, and behavioral deficits following ICH. Accordingly, deferoxamine has been scrutinized for the treatment of secondary brain injury in patients with ICH.

Currently, while deferoxamine is listed as a promising treatment for ICH, there is no validated clinical trial data to confirm the clinical effectiveness of deferoxamine. At present, the following open questions remain and should be interrogated: (1) whether deferoxamine is indeed efficacious in Phase III trials in ICH; (2) although deferoxamine can slow the formation of brain edema and improve resorption of hematoma after ICH (Nakamura et al., 2003), the mechanisms underlying the relationship between the rate of hematoma resorption and the degree of brain edema require further investigation; (3) whether deferoxamine could improve neurological function in patients with ICH when tested at different doses and at earlier treatment initiation times; and (4) whether patients can accept the potential toxic effects of deferoxamine, such as growth retardation or abnormal bone growth, hearing impairment, nephrotoxicity, and other treatment-related adverse events. Further clinical trials should be conducted to control the occurrence of adverse events and to investigate the best therapeutic options that are safe and well tolerated.

## Author Contributions

All authors listed have made a substantial, direct, and intellectual contribution to the work, and approved it for publication.

## Conflict of Interest

The authors declare that the research was conducted in the absence of any commercial or financial relationships that could be construed as a potential conflict of interest.

## Publisher’s Note

All claims expressed in this article are solely those of the authors and do not necessarily represent those of their affiliated organizations, or those of the publisher, the editors and the reviewers. Any product that may be evaluated in this article, or claim that may be made by its manufacturer, is not guaranteed or endorsed by the publisher.
